# From the Basis of Epimorphic Regeneration to Enhanced Regenerative Therapies

**DOI:** 10.3389/fcell.2020.605120

**Published:** 2021-01-21

**Authors:** Béryl Laplace-Builhé, Sarah Bahraoui, Christian Jorgensen, Farida Djouad

**Affiliations:** ^1^IRMB, Univ Montpellier, INSERM, Montpellier, France; ^2^CHU Montpellier, Montpellier, France

**Keywords:** neural crest cell, mesenchymal stem cell, cell therapy, osteoarthritis, regeneration

## Abstract

Current cell-based therapies to treat degenerative diseases such as osteoarthritis (OA) fail to offer long-term beneficial effects. The therapeutic effects provided by mesenchymal stem cell (MSC) injection, characterized by reduced pain and an improved functional activity in patients with knee OA, are reported at short-term follow-up since the improved outcomes plateau or, even worse, decline several months after MSC administration. This review tackles the limitations of MSC-based therapy for degenerative diseases and highlights the lessons learned from regenerative species to comprehend the coordination of molecular and cellular events critical for complex regeneration processes. We discuss how MSC injection generates a positive cascade of events resulting in a long-lasting systemic immune regulation with limited beneficial effects on tissue regeneration while in regenerative species fine-tuned inflammation is required for progenitor cell proliferation, differentiation, and regeneration. Finally, we stress the direct or indirect involvement of neural crest derived cells (NCC) in most if not all adult regenerative models studied so far. This review underlines the regenerative potential of NCC and the limitations of MSC-based therapy to open new avenues for the treatment of degenerative diseases such as OA.

## Introduction

Epimorphic regeneration is a process allowing some vertebrates to regrow complete functional appendages after previous amputation. Adult mammals are not able to regenerate their limbs after injury. However, few vertebrates such as anurans can regenerate their tissues during early development before their metamorphosis and others such as teleost can regrow appendages throughout life. The teleost group presents many specimens able to regenerate. Among them, the zebrafish, *Danio rerio*, is able to regenerate their fins throughout its life, and is a relevant model at adult and larva stages to study this process (White et al., 1994; Marques et al., 2019). In most mammalians, the regenerative potential is tightly limited to some species like the African spiny mouse *Acomys* or the MRL mouse that exhibit enhanced regenerative abilities (Clark et al., 1998; Seifert et al., 2012). However, young humans and adult mice present regenerative abilities after digit tip amputation (Choi et al., 2014).

Eventually, the loss of this regenerative ability and aging will lead to osteoarticular degenerative diseases such as osteoarthritis. Until now, the only treatments used in this context are directed at relieving symptoms including the pain and the decrease of mobility. These last years, efforts in the field of cellular therapy have been performed to bring new outcomes for the treatments. Numerous clinical assays have been conducted using mesenchymal stem cells (MSC) and showed their capacity to regulate the inflammatory response in patients with severe osteoarthritis among others. Indeed, MSC injection induces an immediate local immune response by releasing paracrine factors and cytokines that could generate a cascade of events resulting in a long-lasting systemic immune regulation (Pers et al., 2016, 2018). Thus, phase I and II clinical studies have shown that MSC-based therapy in osteoarticular diseases such as osteoarthritis (OA) is safe and well-tolerated but the joint function is not fully restored in the long term (Pers et al., 2016, 2018; Cosenza et al., 2017; Borakati et al., 2018; Iijima et al., 2018). This absence of long-term therapeutic effect mediated by MSC in OA patients might be due to limitations encountered by MSC related to their intrinsic properties and/or the pathological environment they are exposed to. The heterogeneity of MSC populations associated with their phenotypic, metabolic, and functional instabilities has been also suspected to have greatly contributed to limit their success in OA therapy (Djouad et al., 2005; Isern et al., 2014; Liu et al., 2019).

An emergent explanation of this heterogeneity is the dual embryonic origin of MSC: one source is the mesoderm and the other is the neural crest (Sowa et al., 2013; Isern et al., 2014). This latter MSC source is well-known to give rise to multiple derivatives during development including peripheral nervous system and skeletal elements, which make them an interesting source of MSC in the context of osteoarticular diseases.

The epimorphic regeneration process is well-known to be dependent of the innervation and in particular of the neural crest derived cells (Brockes, 1987; Kumar and Brockes, 2012; Johnston et al., 2016). In order to have a better understanding of the mechanisms that could stimulate joint tissue regeneration, it is important to decipher first the molecular and cellular processes responsible for tissue and organ regeneration in integrated models. Among them, models of regenerative species can provide significant clues and point an overview of this complex phenomenon.

In this review, we will discuss the current problems encountered with MSC-based therapies in the context of OA. We will question their limitations while reviewing their regenerative abilities through the release of trophic factors and their adaptive response to the inflammatory microenvironment. Nonetheless, MSC regenerative abilities may diverge with their heterogeneity highlighting the importance of their embryonic origins with a focus on the neural crest (NC) source known to play a critical role during regeneration in regenerative models.

We will discuss the ability of cells derived from NC to orchestrate a regenerative response by coordinating molecular and cellular events. We will also tackle the importance of unraveling the mystery of regeneration in regenerative species to overcome the mammalian limitations. Overall, this review aims to approach one pending question: how we can properly activate/enhance the regenerative potential in the context of osteoarticular diseases?

## Mesenchymal Stem Cell-Based Regenerative Medicine for Osteoarthritis

### Osteoarthritis and Mesenchymal Stem Cell-Based Therapy

Osteoarthritis (OA) is the most common degenerative and inflammatory joint disorder (Pers et al., 2015). Despite the increase in the incidence of OA, there is still no effective pharmacotherapy capable of restoring the original structure and function of damaged articular cartilage. Consequently, cell-based therapies for OA have become thriving areas of research. Mesenchymal stromal/stem cell (MSC) have turned into the most extensively explored new therapeutic product. In the early 1990s, these cells have attracted a great interest for regenerative medicine. MSC can be isolated and characterized from a wide variety of adult tissues including bone marrow (BM), bone, adipose tissue, synovial membrane, and palatine tonsil, as well as from prenatal structures such as umbilical cord, placenta, and amniotic fluid (Bieback et al., 2004; Djouad et al., 2005; Miao et al., 2006; Janjanin et al., 2008; Maumus et al., 2013). To be referred as MSC, a cell must express a combination of unspecific but characteristic markers including CD73, CD90, and CD105 and negative for markers of hematopoietic stem cells (Dominici et al., 2006). Moreover, MSC are multipotent cells able to differentiate into cells of various cell lineages including adipocytes, chondrocytes, and osteoblasts (Dominici et al., 2006). Because of their relative ease accessibility and isolation associated with their self-renewal potential and multipotency, MSC have been, for decades, candidates of choice for a wide range of clinical applications. MSC mainly exert their regenerative properties through the secretion of bioactive factors that have potent anti-apoptotic, anti-fibrotic, and anti-inflammatory effects (Le Blanc and Ringden, 2006; Djouad et al., 2009a; Caplan and Correa, 2011).

Our understanding of the regenerative properties of MSC has been, in part, improved thanks to the use of experimental mouse models of degenerative diseases. These studies have notably highlighted that the intra-articular (IA) injection of murine MSC reduces synovial thickening, osteophyte formation, and cartilage destruction in experimental OA (ter Huurne et al., 2012; Diekman et al., 2013; Schelbergen et al., 2014). Then, large number of phase I or II clinical trials has thus been launched and has shown that IA MSC injection is safe and well-tolerated (for reviews see Pers et al., 2015; Wang et al., 2019). However, while IA MSC injection has shown improvements in pain and function of OA knees at short-term follow-up, their efficacy on long-term clinical outcomes and cartilage regeneration remains unreported (Ha et al., 2019).

Within a damaged tissue, MSC release trophic factors allowing them to communicate with and educate the surrounding cells. In musculoskeletal applications, MSC therapeutic potential leans on the trophic activities of both administrated exogeneous MSC and the resident cells they might empower within the joint space. The secretome of MSC exposed to an inflammatory microenvironment not only protects the phenotype and the functions of mature and progenitor cells within the joint such as chondrocytes but also potently regulate the immune response (Djouad et al., 2009a; Maumus et al., 2013; Hofer and Tuan, 2016). Indeed, MSC exert potent and well described immunoregulatory properties but not in their naïve state. Rather, stimulation with pro-inflammatory cytokines reveals their immunosuppressive potential that acts in a dose-dependent manner and through both contact-dependent mechanisms and soluble factors (Djouad et al., 2003).

### Mesenchymal Stromal/Stem Cell Regenerative Properties

Given the complexity of the regeneration process, multipotent MSC themselves with poor engraftment and limited survival rates cannot give rise to a fully renewed, well-structured and functional complex tissue (for review see Pittenger et al., 2019). Once in the injured tissue microenvironment, MSC start to release some factors such as programmed cell death 1 ligand 1 (PD-L1), prostaglandin E2 (PGE2), interleukin (IL)-10 and IL-6, and increase their indoleamine 2,3-dioxygenase (IDO) activity (human MSC) or nitric oxide (NO) production (mouse MSC) (Aggarwal and Pittenger, 2005; Spaggiari et al., 2008; Djouad et al., 2009a; Nemeth et al., 2009; Bouffi et al., 2010; Luz-Crawford et al., 2012; Gu et al., 2013). Altogether, these factors are key mediators for MSC immunoregulatory potential (Djouad et al., 2009a). Thus, MSC are highly plastic cells that might adopt either a pro- or an anti-inflammatory response according their inflammatory environment to potently regulate the immune response within the injured tissue (Waterman et al., 2012; Khedoe et al., 2017; Cassano et al., 2018; Avery et al., 2019). Whether this immunoregulatory plasticity is restricted to a particular subset of MSC and variable between MSC donors and tissue source has never been investigated. The identification and characterization of a particular immunoregulatory subset of MSC will significantly improve MSC-based therapy for degenerative diseases exhibiting deleterious inflammatory effects.

MSC also enhance tissue repair by producing trophic factors that promote angiogenesis and empower endogenous cell proliferation, functionality, and differentiation (Caplan and Dennis, 2006). The capacity of MSC to release trophic factors can be enhanced under specific culture conditions that differ from the pro-inflammatory stimulation well-described in the context of MSC immunomodulatory properties. Indeed, MSC cultured under hypoxia exhibit an enhanced capacity to produce trophic factors such as HIF-1α and HGF promoting the release of pro-angiogenic, anti-apoptotic, and anti-fibrotic molecules (Liew and O'Brien, 2012). In the deleterious environment of degenerative OA, enhanced HIF1 expression level is pivotal for chondrocyte survival (Pfander et al., 2006). Moreover, the anti-hypertrophic and anti-fibrotic potential of MSC on OA articular chondrocytes has been shown to be partly mediated by the production of HGF (Maumus et al., 2013).

The heterogeneous clinical efficacy of MSC in human OA might be, in part, associated to the variable inflammatory microenvironment that MSC encounter once injected in OA knees. During OA development, chronic inflammation has been attributed to a self-perpetuating cycle of local damage, inflammation, and repair leading to the comparison of OA joint with a chronic wound (Scanzello et al., 2008). Indeed, joint damage leads to the production of extracellular matrix (ECM) breakdown products that activate fibroblasts-like synoviocytes (FLS), synovial macrophages, and chondrocytes that produce locally inflammatory mediators. This local inflammation promotes cartilage degradation amplifying the vicious cycle of innate immune activation in OA (Sokolove and Lepus, 2013). Thus, inflammation is persistent in OA and the inflammatory signals might vary according the cells activated within the OA joint.

This perpetual inflammatory microenvironment can be also deleterious for MSC properties and even worse can pejoratively activate them in pro-inflammatory MSC similarly to FLS that induce the local production of inflammatory mediators in response to ECM breakdown products. Moreover, pro-inflammatory cytokines such as TNF-β repress MSC osteogenic differentiation through the activation of NF-κB phosphorylation and NF-κB-regulated gene products associated with inflammatory and degradative processes (Constanze et al., 2020). Regarding the chondrogenic potential of MSC, deleterious effects of IL-1 and TNFα have been demonstrated (Majumdar et al., 2001; Djouad et al., 2009b).

The heterogeneity of MSC subpopulations and its consequence on MSC-based therapy has been tackled recently. Indeed, some authors have considered whether among the heterogenous MSC population they are some subsets of MSC with a higher regenerative potential and whether particular subsets are more regenerative whatever the damaged tissue we are studying (O'Connor, 2019). Thus, using single cell RNA sequencing method, MSC heterogeneity has been intensively studied and the existence of several distinct MSC subsets that possess diverse functions has been shown (Huang et al., 2019; Rennerfeldt et al., 2019; Sun et al., 2020). More advances on the identification and characterization of such MSC regenerative subsets are needed for the identification of the most suitable MSC population for a given application and thus significantly improve MSC-based therapy for OA.

### Sex-Dependent Differences in Mesenchymal Stem Cells Biology and in OA Occurrence and Severity

Sex-dependent differences on MSC biology have been intensively studied. First, a sex-dependent MSC transcriptome and secretome was reported (Zeller et al., 2009; Bianconi et al., 2020). Genes associated to several processes such as differentiation, inflammation, and cell communication are differentially expressed in female and male MSC (Bianconi et al., 2020). Indeed, MSC differentiation is sexually dimorphic and might be influenced by different factors including genetic factors in the case of osteogenesis (Zanotti et al., 2014). Also, MSC derived from rat female secrete more growth factors and less proinflammatory cytokines than their male counterpart (Zeller et al., 2009). These discrepancies between female and male MSC could significantly impact the clinical outcome assessment underlying that the donor sex should be considered for proper and optimal use of MSC in clinic.

Moreover, although complex in the pathogenesis of OA, the role of endogenous sex hormones and reproductive factors on OA of the hip, knee, and hand has been evidenced (Hussain et al., 2018). Thus, differences in terms of severity and occurrence of OA have been reported. Main sex differences have been found at the level of synovial inflammation, cartilage degradation, osteophyte formation, subchondral bone deterioration, and pain (Javaheri et al., 2018; Sannajust et al., 2019). For example, in rats, a more severe inflammation of the synovial membrane as well as a more intense degradation of the cartilage and subchondral bone have been observed in females as compared to males (Javaheri et al., 2018). This exacerbated severity in females is associated with a more important infiltration of macrophages positive for CD68 in the synovial membrane. Sex-dependent differences in severity have also been observed in a large animal model such as in pig females that exhibit worse biomechanical outcomes and more important cartilage damage than males (Kiapour et al., 2015a,b,c). In humans, OA incidence is also sexually dimorphic. Indeed, after the age of 50, women have a higher rate of OA than men. Research has investigated the contribution of sex hormones, reproductive factors, and hormone supplementation to osteoarthritis (Contartese et al., 2020). Although complex in the pathogenesis of OA, the role of endogenous sex hormones and reproductive factors on OA of the hip, knee, and hand has been evidenced (Hussain et al., 2018).

### Mesenchymal Stem Cell Embryonic Origin

Despite the promising results of MSC-based therapy to treat degenerative diseases such as OA in preclinical models, the identification of a molecular signature to isolate a specific homogeneous population of MSC that would meet regenerative medicine needs is still pending. MSC are currently used in clinic to induce tissue repair, however, the repaired tissue is structurally different to the native tissue and non-functional. This might be due to the phenotypic and functional heterogeneity of MSC that exhibit a mixed ontogeny. Although mesenchymal tissues derive from different developmental origins, the cranial NC for the face, and the mesoderm for the trunk, it has long been thought that MSC derive from mesoderm. Over these last two decades, many questions in the field of MSC has revolved around their origin. Takashima and colleagues have shown, using Sox1-Cre/YFP transgenic midgestational mouse embryos, that MSC can be isolated from trunk neuroepithelial cells while they cannot from mesodermal cells (Takashima et al., 2007). In contrast, in post-natal BM, they found that neuroepithelium/NC-derived MSC were still present but to a lower extent (Takashima et al., 2007). Thus, the authors conclude that during the development a first wave of MSC derives from the neuroepithelium and NC followed by a second wave of MSC from an unknown origin that emerges after birth. More recently, using Nestin/GFP;Wnt1Cre2;R26/Tomato transgenic mice, the existence of ontogenically distinct MSC was confirmed with evidence for functional disparities between MSC derived from different germ layers (Isern et al., 2014). Indeed, MSC positive for Nestin that form the niche of HSC in perinatal BM originate from NC and modestly contribute to endochondral ossification. These cells preserve their MSC activity after birth. In contrast, MSC negative for Nestin derived from the mesoderm are mainly involved in fetal endochondrogenesis and do not maintain their activity after birth (Isern et al., 2014). Together these studies suggest the transient co-existence of MSC with different embryonic origins within the bone marrow during the development and that NC-derived MSC retaining their activity after birth might participate to the physiological turnover of the skeleton in adults (**Figure 2**).

These findings suggest that the heterogeneous clinical efficacy of MSC in human OA might be, in part, associated to the variable proportion of MSC derived either from the mesoderm or neural crest within the BM or the other sources of MSC. Moreover, the findings made by Isern and colleagues clearly demonstrating that active NC-derived MSC are still present in the BM of mammals after birth (Isern et al., 2014) raise the following questions: Why are NC-derived MSC not recruited to the injured and degraded OA joint to activate the regeneration process? Can exogeneous NC-derived MSC be more efficient to treat OA?

In line with this hypothetical enhanced regenerative potential of MSC derived from NC, it is now well-accepted that epimorphic regeneration in regenerative models relies on neural crest cells. Indeed, neural crest-derived nerve mesenchymal cells participate to bone and dermis formation during digit tip regeneration in mammals (Carr et al., 2018). Thus, a better understanding of how neural crest derived cells are involved in regeneration will open up a whole new avenue for degenerative disease treatment.

## Limb Regeneration in Vertebrates

**Figure 1:**
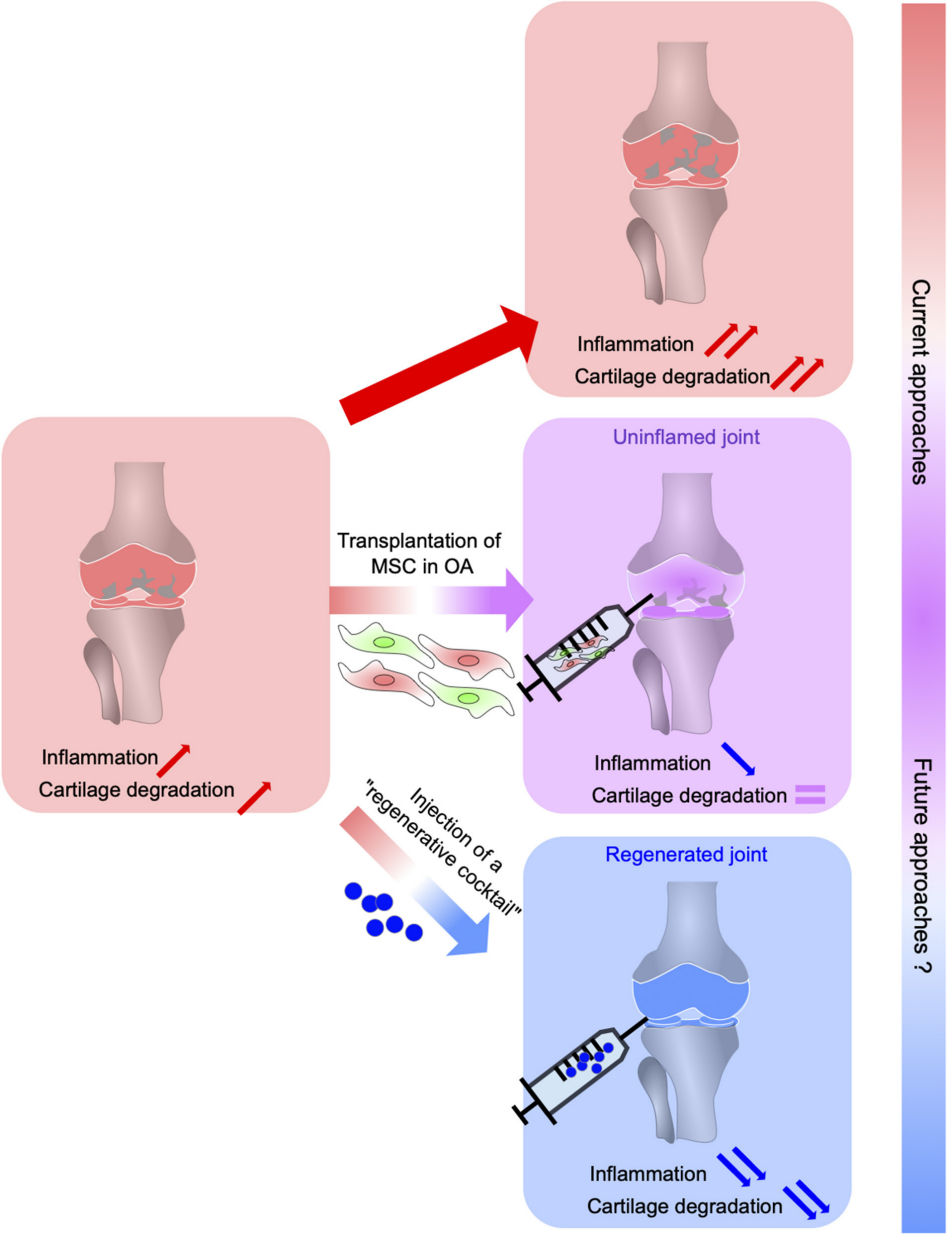
From MSC-based suppression of immune response in OA to the regeneration of OA joints using a cocktail of anti-inflammatory and regenerative factors (regenerative cocktail). Osteoarthritis (OA), the most common inflammatory and degenerative joint disease, is complex and multifaceted characterized by cartilage degradation, subchondral bone changes, osteophytes formation, and synovium inflammation. MSC administration suppresses immune cell migration, activation, and production of inflammatory cytokines leading to a reduced inflammation in OA joints without allowing joint tissue regeneration. The development of novel therapeutic strategies to both regulate the immune response and induce tissue regeneration leans on a better understanding of the regeneration process possible in some vertebrates. This relies on the identification of the mechanisms underlying regeneration that will determine key factors that control inflammation while orchestrating stem cell proliferation, differentiation, and tissue regeneration.

As has been discussed earlier in this review, a therapy in OA based on “regenerative” factors, rather than MSC based therapy, will likely be used in the future (Figure 1). Moreover, other cell sources than MSC or MSC derived from NC should be considered and studied on their secretome and will undoubtedly help to identify key molecules that govern regeneration. To identify such cells that govern regeneration and the factors they release, numerous studies now focus on vertebrate species able to regenerate their limbs, and on the discovery of endogenous factors that explain this rare ability. This mechanism is called epimorphic regeneration and relies on several steps. After amputation there is a reepithelization of the wound, which will lead to the formation of a specialized structure called “apical epithelial cap” or AEC. Then, there is the formation of a particular structure called the blastema, with a peak of proliferation. The last step consists in repatterning to obtain a fully formed limb theoretically identical to the former one. Few vertebrates are capable of such process, among them the famous axolotl and salamander, which regenerate limbs during all their life, but also teleost fishes such as Zebrafish. Some amphibians such as Xenopus are able to regenerate but only during the very first steps of development and lose their abilities after metamorphosis. The blastema composition is still debated, nevertheless the current consensus is that it is formed by an heterogenous mass of cells, mainly dedifferentiated cells from injured or surrounding tissues, entering the cell cycle. The reason why mammals in their vast majority cannot regenerate appendages at adult stage, or form a blastema, is unknown. Lately, at least two systems have been described for their involvement in blastema formation and overall regeneration: the immune system and the nervous system. These systems will be tackled in further details below.

Among immune cells, macrophages have particularly triggered scientist attention in the context of regeneration (Chazaud, 2014; Wynn and Vannella, 2016). In salamander, macrophages, monocytes, and granulocytes are detected since 1-day post amputation. They accumulate very fast and their early depletion completely inhibits the regeneration process, whereas a late depletion only delays it (Godwin et al., 2013). In zebrafish, macrophages also play a crucial role during fin regeneration in adult and larvae (Petrie et al., 2014; Nguyen-Chi et al., 2015, 2017). As in salamander, early recruited macrophages are important for blastema formation and the late ones more involved in tissue remodeling. Likewise, adult mouse, macrophage involvement during digit tip regeneration has been described. These cells are necessary to the whole process: from the AEC formation to the repatterning of the appendage (Simkin et al., 2017). If the importance of these cells has been widely described, their effector functions are less known in the context of regeneration. Because of their plasticity and their abilities to polarize in response to the environment, recent studies have been focused on inflammatory mediators released by macrophages and their functions during regeneration. The zebrafish mutant *cloche*, which has no hematopoietic tissue, is not able to regenerate (Hasegawa et al., 2015). The first step of wound healing is conserved but there is no proliferation of blastema cells, and extensive apoptosis. To understand if a diffusible factor produced by hematopoietic cells could be responsible for maintenance and proliferation of blastema cells, the authors co-cultured mutant fin explants with wild type (WT) or mutant larva extracts. Proliferation rescue happened in the co-cultures with WT larva extract, suggesting the existence of hematopoietic derived diffusible factors (Hasegawa et al., 2015). Next, the authors showed an IL-1β abnormal expression triggering long lasting and uncontrolled inflammatory phase. Similarly, macrophage depletion triggered aberrant IL-1β overexpression. Moreover, in intact larvae, an overexpression of IL-1β triggered ectopic expression of some blastemal markers (Hasegawa et al., 2017). Furthermore, we have shown the crucial role of TNFa/TNFR1 axis mediated by macrophages during caudal fin regeneration, which induces blastema proliferation (Nguyen-Chi et al., 2017). These studies show that macrophages likely adjust the inflammatory response during the regeneration.

Other teams have investigated the inflammatory modulation role during regeneration, showing that inflammation blockade is deleterious for the regeneration process (Mathew et al., 2007; Kyritsis et al., 2012; Li et al., 2012). It suggests that inflammation is necessary to the regeneration but needs to be fine-tuned. However, the mechanisms by which macrophages are recruited and controlled remain unknown. The identification of such mechanisms is critical to determine the role of an appropriate macrophage response in the context of degenerative mammalian diseases such as OA. Since regeneration medicine is an integrated process, the use of regenerative animal models for an integrated vision of regeneration is highly informative not only to identify all the cellular components required for the formation of the blastema but also to determine how these components communicate and regulate each other to give rise to a regenerated tissue. In this context, the role of the nervous system has been intensively investigated.

### Nerve Dependency During Regeneration Process

The formation of the two crucial structures (e.g., “AEC” and the blastema) for epimorphic regeneration are tightly linked to innervation. That nerve dependency has been discovered by Todd in 1823 who has observed an impaired limb regrowth after sciatic nerve transection (Todd, 1823; Dinsmore and Solomon, 1991). Later, Singer established the “neurotrophic hypothesis” based on experiments in amphibians and in chick embryos (Singer, 1952; Geraudie and Singer, 1981; Fowler and Sisken, 1982). This theory has been widely accepted and applied to other models to identify trophic factors secreted by the nerve and responsible for the maintenance of the AEC and the blastema subsequently. One useful model developed in the salamander is the “accessory limb model” (Endo et al., 2004, 2015), which allows to isolate more easily the sufficient and necessary neurotrophic factors secreted during regeneration. This model consists in the brachial nerve deviation to a skin wound on the limb side to induce the formation of an ectopic blastema-like structure. If this manipulation is followed by a graft from the epidermis, at proximity of the injury, the blastema formation leads to the formation of an entire accessory limb, demonstrating the tight cooperation between AEC-blastema and the nerve. Moreover, if the wound healing is not dependent of the nerve, the formation of the AEC that allows the blastema establishment is nerve dependent. Interestingly, the peripheral nerve system arises from a structure called the neural crest (NC). These neural crest cells (NCC) intrigued for a long-time developmental biologists as they give rise to a broad number of derivatives but also because of their plasticity.

### Neural Crest Cells

The neural crest cells (NCC) are a transient embryonic cell population emerging during neurulation in vertebrates, discovered by (His, 1868) and following experiments conducted by Höstradius with ablation of NC to observe their contribution to organ/part of the body formation (Le Douarin, 2004). NCCs arise from the folding of neural plate into the neural tube orchestrated by a fine-tuned gene regulatory network (GRN) composed of different groups participating to successive events leading to their final fate (Sauka-Spengler and Bronner-Fraser, 2008). The first is their induction with factors secreted from mesoderm and notochord such as signaling pathway molecules including bone morphogenetic proteins (BMP), WNT, Sonic Hedgehog (SHH), fibroblast growing factor (FGF), and Retinoic Acid (RA). Then, their spatial definition takes place with neural-plate border defining molecules (Zic1, Msx1/2, Pax3/7, and Ap2), which will allow NCCs to progress toward their specification with expression of characteristic markers (FoxD3, Sox10, and Snail). In the neural-plate border, specific signals induce a complex transcription factor network that will control NC specification and epithelial-to-mesenchymal transition (EMT). After this transition from an epithelial to a mesenchymal phenotype, NCC acquire a migratory phenotype and migrate through the cranial and trunk regions of the embryo (see for review Green et al., 2015). Considered as a key feature in vertebrate evolution thanks to their morphological contribution, NCC GRN is conserved across them but can differ slightly as certain transcription factors may be crucial to their formation or not. Although cell types derived from NCCs are found in invertebrates, most important evolutionary aspects are cranial elements, glia, neurons, and pigment cells (Le Douarin et al., 2004; Bronner and LeDouarin, 2012).

### Neural Crest Cells Multipotency

NCCs give rise to many derivatives and their migratory potential allows their presence in virtually all the tissues (Trainor, 2005). However, the question of their multipotency has remained elusive. The NCCs have been defined either as a heterogeneous population containing restricted progenitors, which fate is determined early during the development, or as a multipotent cell population (Dupin and Sommer, 2012; Le Douarin and Dupin, 2016). Some evidences have been proposed in favor of the first hypothesis in the chick embryo. This model proposed that NCC were determined from the emergence of the crest, according to their position within the neural tube. Recently, a study investigated this point, using a new transgenic line (Baggiolini et al., 2015) has revealed that pre-migratory and migratory NCC are multipotent. They have thus resolved this controversy using *in vivo* cell fate mapping of trunk NCC with the R26R-Confetti mouse and demonstrated that the majority of pre-migratory and migratory NCC are multipotent. In another model, the *Xenopus Larvae*, the expression of pluripotency factors at blastula stage and neurula stages was studied (Buitrago-Delgado et al., 2015). They showed that NCC derived from these selected blastula cells with pluripotency characteristics have the ability to retain and maintain them along their evolution (Buitrago-Delgado et al., 2015).

FGF, Wnt1, or BMP signaling pathways are activated during the induction of NCCs. Thus, NCC pluripotency was at first accredited to a gain of function phenomenon following their exposure to molecules of FGF, Wnt1, or BMP signaling pathways. However, more recently, in Xenopus, it has been demonstrated that NCC arise from a subset of pluripotent cells that has retained FGF-mediated Map Kinase signaling (Geary and LaBonne, 2018).

Importantly, multipotent NCC have also been identified in the neonatal and adult mammals (Motohashi et al., 2011, 2014; Kunisada et al., 2014; Motohashi and Kunisada, 2015). Indeed, they have been identified in the adult rat palatum, periodontal ligament (Techawattanawisal et al., 2007; Widera et al., 2007, 2009), and in the dental pulp tissue (Sasaki et al., 2008). Moreover, multipotent cardiac multipotent NCC have been identified. This latter cells form spheres *in vitro*, present NCC characteristics and differentiate, in addition to cardiomyocytes, into NC derivatives such as peripheral nervous system neurons, glial cells (Tomita et al., 2005).

### Neural Crest-Derived Stem Cells During Tissue Repair in Adult Mammals

**Figure 2:**
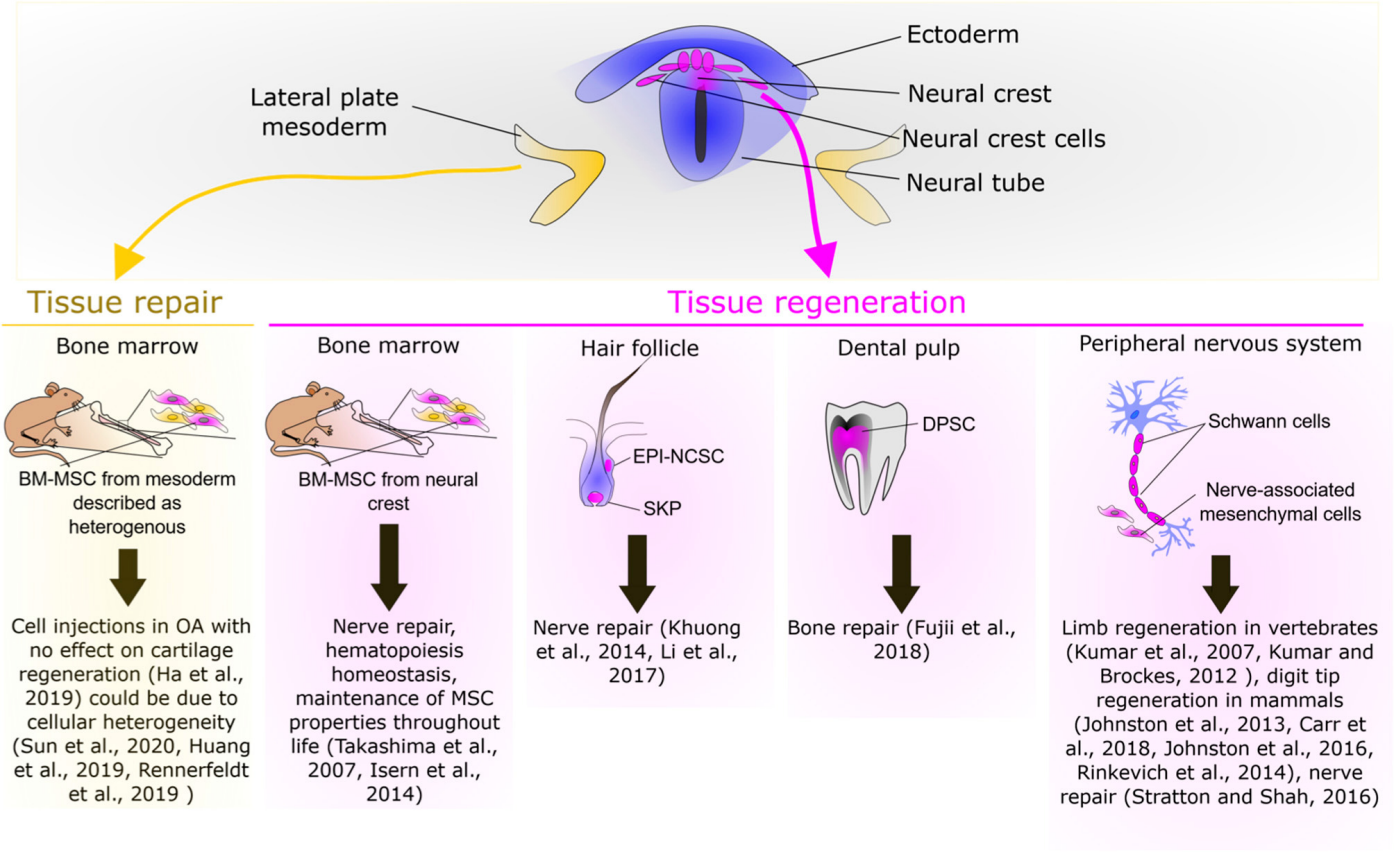
Overview of the repair or regenerative potential of neural crest cells.

Neural crest-derived stem cells (NCSC) are not restricted to the embryonic NC, but they are also present in several NC-derived tissues in adult vertebrates. Some post-migratory NCSC have conserved similarities with their embryonic counterparts such as their capacity to differentiate into a large variety of cell types. Moreover, these cells have been described for their role in the context of tissue repair (Figure 2), in part, through their capacity to regulate the immune response. Indeed, in the defected sciatic nerve of rats, epidermal-neural crest stem cells (EPI-NCSC) was shown to promote the recovery of structure and function of nerve bridged with artificial nerve by regulating inflammation and providing a supportive microenvironment for the defected nerve repair (Li et al., 2017). Similarly, transplantation of another NCSC population named olfactory ensheathing cell (OEC) has been described to improve and promote axon regeneration after a complete low-thoracic spinal cord transection in adult rats, through neuroprotective and immunomodulatory mechanisms that create a permissive environment (Khankan et al., 2016). This tissue repair potential has been extended to other NCSC such as the skin-derived precursors pre-differentiated into Schwann cells (SKP-SC). Indeed, following sciatic nerve injury SKP-SC administration significantly improves the mean axon counts and behavioral recovery (Khuong et al., 2014). Finally, the use of other NCSC such as dental pulp stem cells (DPSC) and inferior nasal turbinate stem cells (ITSC) should be further considered for bone repair given their capacity to generate into osteoblasts (Schurmann et al., 2014; Fujii et al., 2018; Amghar-Maach et al., 2019). Regarding periodontal regeneration a comparative study has shown that periodontal ligament stem cells exhibit a higher regenerative potential than DPSC (Amghar-Maach et al., 2019). Altogether, these studies underline the potent regenerative capacity of NCSC, in part, through the regulation of the immune response. This potential should be further investigated in comparative studies in order to identify the optimal NCSC source for a given therapeutic application.

### Schwann Cells in Regenerative Models

As we mentioned, regeneration relies on innervation and in particular on nerve associated cells such as neural crest derived cells including Schwann cells (SC) or Schwann cell precursors (SCP). In the context of adult skin repair in mice, SC contribute to dermis regeneration in a Sox-2 dependent manner (Johnston et al., 2013). In newts and salamander, during epimorphic regeneration, SC accumulate in the regenerating limb and produce factors sufficient for blastema cell proliferation and regeneration of distal structures (Kumar et al., 2007; Kumar and Brockes, 2012). Similarly, in mammalian models of regeneration such as murine the digit tip regeneration that depends on nerve (Rinkevich et al., 2014; Johnston et al., 2016), SCP have been shown to dedifferentiate and release growth factors after the removal of the distal digit. Depletion of SCP impaired the proliferation rate of mesenchymal precursors within the blastema and digit regeneration while exogeneous SCP transplantation as well as the local injection of their paracrine factors (oncostatin and platelet-derived growth factor AA) rescued the impairment in digit tip regeneration induced by SCP ablation or denervation (Johnston et al., 2016). Going further, it has recently been shown that injured nerves also contains mesenchymal precursor cells that directly participate to skin and bone formation during digit tip regeneration (Carr et al., 2018). Altogether, these studies demonstrate that in response to digit tip removal, local tissue and nerve lesions induce SC dedifferentiation and release of growth factors that promote neural crest-derived mesenchymal cell proliferation, migration toward damaged tissues, differentiation, and digit tip regeneration (Johnston et al., 2016; Carr et al., 2018).

Interestingly, after peripheral nerve injury, SC secrete several trophic factors that promote macrophage recruitment. In this context, SC recruit pro-inflammatory macrophages and promote their polarization toward an non-inflammatory phenotype (Stratton and Shah, 2016). Thus, SC have been suggested to regulate the inflammatory response. However, this hypothesis has only been explored in the context of peripheral nerve injury. Investigating this aspect in epimorphic regeneration could allow us to better understand the inflammatory regulation and identify crucial factors involved in these regulatory mechanisms.

## Conclusion

This review underlines how the well-recognized heterogeneity of MSC has greatly contributed to limit their success in degenerative disease therapies. Additionally, among MSC subsets ontogenically distinct, there is a need for a molecular characterization of MSC heterogeneity and the identification of a specific phenotype for regenerative MSC that would permit their enrichment during the manufacturing process. As discussed above, this heterogeneity could be related to their dual embryonic origins. From a therapeutic point of view, it is critical to determine which MSC subsets are preferentially expended using the routine protocols for MSC isolation and amplification and compare the regenerative potential of NC-derived MSCs with the ones that derive from the mesoderm in experimental models of degenerative diseases. Moreover, although MSC are potent immunoregulatory cells, inflammation has been shown to be deleterious for certain MSC's functions, among which are their differentiation potential thus limiting their regenerative potential. Thus, whether there is a particular subset of MSC more susceptible to the deleterious effects of inflammation needs to be defined.

Additionally, this review points out the high importance of studying regenerative species in order to comprehend the coordination of molecular and cellular events during this particularly complex phenomenon. Lately, NC-derived cells have emerged as a novel source of pro-regenerative cells. However, the precise mechanisms behind the capacity of these cells to control inflammation and orchestrate regeneration are not fully known and their identification would pave the way for the development of novel therapeutic strategies for degenerative diseases such as OA characterized both by an abnormal immune response and tissue degradation.

Herein, the regenerative models we described could allow us to overcome MSC-based therapy limitations by precisely helping to identify the key events leading to a complete restoration of the injured tissues. The orchestrator role of the NC-derived cells during the regeneration may leads to the discovery of crucial molecular factors allowing to regulate both the inflammatory response and the renewal of the lost tissue (Figure 1). These perspectives open the way for future therapies based on pro-regenerative factors to treat OA.

## Author Contributions

BL-B, SB, CJ, and FD wrote the manuscript. All authors contributed to the article and approved the submitted version.

## Conflict of Interest

The authors declare that the research was conducted in the absence of any commercial or financial relationships that could be construed as a potential conflict of interest.
