# Supplementary material for: From the Basis of Epimorphic Regeneration to Enhanced Regenerative Therapies
Source: Front Cell Dev Biol. 2021 Jan 21;8:605120. doi: 10.3389/fcell.2020.605120 (PMC7873919; doi:10.3389/fcell.2020.605120)
Supplement: Supplementary file 1 [file Data_Sheet_1.PDF]

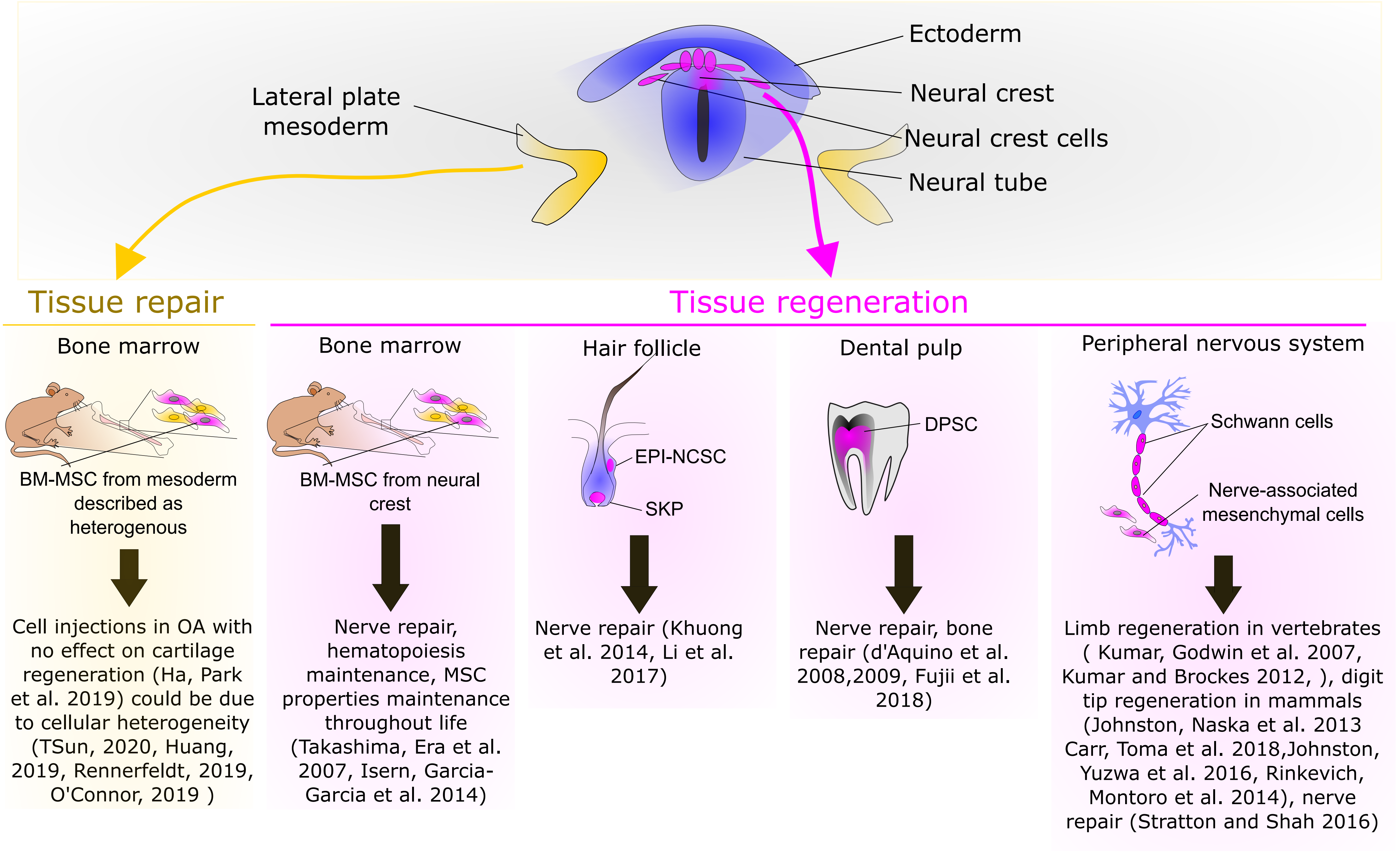

Ectoderm
Neural crest
Lateral plate mesoderm
Neural crest cells
Neural tube
Tissue repair
Tissue regeneration
Bone marrow
BM-MSC from mesoderm described as heterogenous
Cell injections in OA with no effect on cartilage regeneration (Ha, Park et al. 2019) could be due to cellular heterogeneity (TSun, 2020, Huang, 2019, Rennerfeldt, 2019, O'Connor, 2019)
Bone marrow
BM-MSC from neural crest
Nerve repair, hematopoiesis maintenance, MSC properties maintenance throughout life (Takashima, Era et al. 2007, Isern, Garcia-Garcia et al. 2014)
Hair follicle
EPI-NCSC
SKP
Nerve repair (Khuong et al. 2014, Li et al. 2017)
Dental pulp
DPSC
Nerve repair, bone repair (d'Aquino et al. 2008,2009, Fujii et al. 2018)
Peripheral nervous system
Schwann cells
Nerve-associated mesenchymal cells
Limb regeneration in vertebrates (Kumar, Godwin et al. 2007, Kumar and Brockes 2012,), digit tip regeneration in mammals (Johnston, Naska et al. 2013 Carr, Toma et al. 2018,Johnston, Yuzwa et al. 2016, Rinkevich, Montoro et al. 2014), nerve repair (Stratton and Shah 2016)
